# Three new species of *Talaromyces* sect. *Talaromyces* discovered in China

**DOI:** 10.7717/peerj.18253

**Published:** 2024-10-11

**Authors:** Xin-Tong Ren, Saifei Li, Yongming Ruan, Long Wang

**Affiliations:** 1 College of Life Sciences, Zhejiang Normal University, Jinhua, Zhejiang, China; 2 Technology Development and Transfer Center, Institute of Microbiology, Chinese Academy of Sciences, Beijing, China; 3 State Key Laboratory of Mycology, Institute of Microbiology, Chinese Academy of Sciences, Beijing, China

**Keywords:** Molds, New taxa, Polyphasic taxonomy, Soil fungi, Trichocomaceae

## Abstract

**Background:**

*Talaromyces* species play an important role in the nutrient cycle in natural ecosystems, degradation of vegetal biomass in industries and the implications in medicine. However, the species diversity of this genus is still far from fully understood.

**Methods:**

The polyphasic taxonomic approach integrating morphological comparisons and molecular phylogenetic analyses based on *BenA*, *CaM*, *Rpb2* and ITS sequences was used to propose three new *Talaromyces* species.

**Results:**

Three new species of sect. *Talaromyces* isolated from soil are proposed, namely, *T. disparis* (ex-type AS3.26221), *T. funiformis* (ex-type AS3.26220) and *T. jianfengicus* (ex-type AS3.26253). *T. disparis* is unique in low growth rate, velvety texture, limited to moderate sporulation, biverticillate, monoverticillate and irregular penicilli bearing a portion of abnormally large globose conidia, it has no close relatives in phylogeny. Being a member of *T. pinophilus* complex, *T. funiformis* produces mycelial funicles on Czapek yeast autolysate agar (CYA), 5% malt extract agar (MEA) and yeast extract (YES), sparse sporulation on Czapek agar (Cz), CYA, MEA and YES while abundant on oatmeal agar (OA), bearing appressed biverticillate penicilli and globose to pyriform conida with smooth to finely rough walls. *T. jianfengicus* belongs to *T. verruculosus* complex, is characterized by velvety colony texture with moderate to abundant elm-green conidia *en masse*, producing biverticillate penicilli, globose conidia with verrucose walls.

**Conclusion:**

It is now a common practice in establishing new species of *Aspergillus*, *Penicillium* and *Talaromyces* based on morphological characters and phylogenetic analyses of *BenA*, *CaM*, *Rpb2* and ITS sequences. The proposal of the three novelties of *Talaromyces* in this article is not only supported by their morphological distinctiveness, but also confirmed by the phylogenetic analyses of the concatenated *BenA-CaM-Rpb2* and *BenA-CaM*-ITS, as well as the individual *BenA, CaM, Rpb2* and ITS sequence matrices.

## Introduction

*Talaromyces* species are common fungi inhabiting various terrestrial, aquatic (freshwater and marine) and atmospheric environments, which play an important role either in the nutrient cycle of natural ecosystems or the sustainable development of society, for example, producing lignocellulolytic enzymes in plant biomass degradation, synthesizing bioactive secondary metabolites with medical importance, or as the biological control agents in agriculture, *etc*. But some species are opportunistic pathogens causing talaromycosis in humans and animals, such as *T. marneffei* (*e.g*., Yilmaz et al., 2014).

*Talaromyces* species are often encountered and readily recognized on isolating media due to their yellow, orange or reddish colors in the mycelium and/or substratum. However, in contrast to this superficial recognition, species in this genus are less distinguishable from each other by morphological characters in most cases, especially by micro-morphological characters. The molecular approach has to be used in the identification of these fungi. On the basis of four genetic loci, *i.e*., β-tubulin gene (*BenA*), calmodulin gene (*CaM*), DNA-dependent RNA polymerase II second largest subunit gene (*Rpb2*) and the nuclear rDNA ITS1-5.8S-ITS2 (ITS), and the integration with morphological characters, about 208 species have been reported in *Talaromyces*. The genus are currently resolved into eight sections, *i.e*., sections *Bacillispori*, *Helici*, *Islandici*, *Purpurei*, *Subinflati*, *Talaromyces*, *Tenues* and *Trachyspermi* (*e.g*., Samson et al., 2011; Yilmaz et al., 2014; Sun et al., 2020; Houbraken et al., 2020; Lacey et al., 2023; Liu et al., 2023; Nguyen & Lee, 2023; Špetík et al., 2023; Zang et al., 2023; Mo et al., 2024; Visagie et al., 2024; Okubo, Itagaki & Hirose, 2024). Sect. *Talaromyces* is the largest section which now includes about 90 species (Houbraken et al., 2020; Lacey et al., 2023; Liu et al., 2023; Nguyen & Lee, 2023; Visagie et al., 2024) (Table 1).

**Table 1:** The ninety species reported in sect. *Talaromyces*, their ex-types and genetic markers, and the three novelties proposed in this study.

Species	Ex-types	Genetic markes			
		ITS	*Ben A*	*CaM*	*Rpb2*
*T. aculeatus*	NRRL 2129 = CBS 289.48	KF741995	KF741929	KF741975	KM023271
*T. adpressus*	CGMCC 3.18211 = CBS 140620	KU866657	KU866844	KU866741	KU867001
*T. alveolaris*	CBS 142379	LT558969	LT559086	LT795596	LT795597 (antisense)
*T. amazonensis*	CBS 140373 = IBT 23215	KX011509	KX011490	KX011502	MN969186
*T. amestolkiae*	CBS 132696 = DTO 179F5	JX315660	JX315623	KF741937	JX315698
*T. angelicae*	KACC 46611	KF183638	KF183640	KJ885259	KX961275
*T. annesophieae*	CBS 142939	MF574592	MF590098	MF590104	MN969199
*T. apiculatus*	CBS 312.59 = FRR 635	JN899375	KF741916	KF741950	KM023287
*T. argentinensis*	NRRL 28750	MH793045	MH792917	MH792981	MH793108
*T. aspriconidius*	CBS 141835 = DTO 340-F8	MN864274	MN863343	MN863320	MN863332
*T. atkinsoniae*	BRIP 72528a	OP059084	OP087524	N/A	OP087523
*T. aurantiacus*	CBS 314.59 = NRRL 3398	JN899380	KF741917	KF741951	KX961285
*T. aureolinus*	AS3.15865	MK837953	MK837937	MK837945	MK837961
*T. australis*	CBS 137102 = IBT 14256	KF741991	KF741922	KF741971	KX961284
*T. bannicus*	AS3.15862	MK837955	MK837939	MK837947	MK837963
*T. beijingensis*	CGMCC 3.18200 = CBS 140617	KU866649	KU866837	KU866733	KU866993
*T. brevis*	CBS 118436 = DTO 004-D8	MN864269	MN863338	MN863315	MN863328
*T. calidicanius*	CBS 112002	JN899319	HQ156944	KF741934	KM023311
*T. californicus*	NRRL 58168	MH793056	MH792928	MH792992	MH793119
*T. cavernicola*	URM 8448	ON862935	OP672383	OP290543	OP290515
*T. cnidii*	KACC 46617	KF183639	KF183641	KJ885266	KM023299
*T. coprophilus*	CBS 142756	LT899794	LT898319	LT899776	LT899812 (antisense)
*T. cucurbitiradicus*	ACCC 39155 = CGMCC 3.26140	KY053254	KY053228	KY053246	OR242024 (CN090C2)*
*T. derxii*	CBS 412.89 = NHL 2981	JN899327	JX494306	KF741959	KM023282
*T. dimorphus*	AS3.15692 = NN072337	KY007095	KY007111	KY007103	KY112593
*T. domesticus*	NRRL 58121	MH793055	MH792927	MH792991	MH793118
*T. duclauxii*	CBS 322.48 = NRRL 1030	JN899342	JX091384	KF741955	JN121491
*T. echinulatus*	CNUFC HB1206	OR462362	OR507571	OR608367	OR591610
*T. euchlorocarpius*	DTO 176I3 = CBM PF1203	AB176617	KJ865733	KJ885271	KM023303
*T. flavovirens*	CBS 102801 = IBT 27044	JN899392	JX091376	KF741933	KX961283
*T. flavus*	CBS 310.38 NRRL 2098	JN899360	JX494302	KF741949	JF417426
*T. francoae*	CBS 113134 = IBT 23221	KX011510	KX011489	KX011501	MN969188
*T. funiculosus*	CBS 272.86 = IMI 193019	JN899377	JX091383	KF741945	KM023293
*T. fuscoviridis*	CBS 193.69 = IBT 14846	KF741979	KF741912	KF741942	MN969156
*T. fusiformis*	CGMCC 3.18210 = CBS 140637	KU866656	KU866843	KU866740	KU867000
*T. galapagensis*	NRRL 13068 = CBS 751.74	JN899358	JX091388	KF741966	MH793105
*T. ginkgonis*	CGMCC 3.20698	OL638158	OL689844	OL689846	OL689848
*T. haitouensis*	AS3.16101	MZ045695	MZ054634	MZ054637	MZ054631
*T. indigoticus*	CBS 100534 = IBT 17590	JN899331	JX494308	KF741931	KX961278
*T. intermedius*	CBS 152.65 = IMI 100874	JN899332	JX091387	KJ885290	KX961282
*T. kabodanensis*	CBS 139564 = DTO 204-F2	KP851981	KP851986	KP851995	MN969190
*T. johnpittii*	BRIP 72504a = MST-FP2594	OP712677	OP712647	OP712645	OP712646
*T. kendrickii*	CBS 136666 = IBT 13593	KF741987	KF741921	KF741967	MN969158
*T. lentulus*	AS3.15689	KY007088	KY007104	KY007096	KY112586
*T. liani*	CBS 225.66 = NRRL 3380	JN899395	JX091380	KJ885257	MH793100
*T. louisianensis*	NRRL 35823	MH793052	MH792924	MH792988	MH793115
*T. macrosporus*	CBS 317.63 = FRR 404	JN899333	JX091382	KF741952	KM023292
*T. mae*	AS3.15690	KY007090	KY007106	KY007098	KY112588
*T. malicola*	NRRL 3724	MH909513	MH909406	MH909459	MH909567
*T. mangshanicus*	AS3.18013	KX447531	KX447530	KX447528	KX447527
*T. marneffei*	CBS 388.87 = IMI 068794ii	JN899344	JX091389	KF741958	KM023283
*T. muroii*	CBS 756.96 = PF 1153	MN431394	KJ865727	KJ885274	KX961276
*T. mycothecae*	CBS 142494	MF278326	LT855561	LT855564	LT855567
*T. nanjingensis*	JP-NJ4 = M 2012167	MW130720	MW147759	MW147760	MW147762
*T. neofusisporus*	AS3.15415 = CBS 140623	KP765385	KP765381	KP765383	MN969165
*T. oumae-annae*	CBS 138208 = DTO 269-E8	KJ775720	KJ775213	KJ775425	KX961281
*T. panamensis*	CBS 128.89 = IMI 297546	JN899362	HQ156948	KF741936	KM023284
*T. penicillioides*	AS3.15822	MK837956	MK837940	MK837948	MK837964
*T. pinophilus*	CBS 631.66 = IMI 114933	JN899382	JX091381	KF741964	KM023291
*T. pratensis*	NRRL 62170	MH793075	MH792948	MH793012	MH793139
*T. primulinus*	CBS 321.48 = NRRL 1074	JN899317	JX494305	KF741954	KM023294
*T. pseudofuniculosus*	CBS 143041	LT899796	LT898323	LT899778	LT899814 (antisense)
*T. purgamentosus*	CBS 113145	KX011504	KX011487	KX011500	MN969189
*T. purpureogenus*	CBS 286.36 = IMI 091926	JN899372	JX315639	KF741947	JX315709
*T. qii*	AS3.15414 = CBS 139515	KP765384	KP765380	KP765382	MN969164
*T. rapidus*	CBS 142382 = UTHSC DI 16-148	LT558970	LT559087	LT795600	LT795601
*T. ruber*	CBS 132704 = DTO 193H6	JX315662	JX315629	KF741938	JX315700
*T. rubicundus*	CBS 342.59 = NRRL 3400	JN899384	JX494309	KF741956	KM023296
*T. rufus*	CBS 141834 = CGMCC 3.13203	MN864272	MN863341	MN863318	MN863331
*T. sayulitensis*	CBS 138204 = DTO 245H1	KJ775713	KJ775206	KJ775422	MH793141 (NRRL 62185)*
*T. shilinensis*	CGMCC 3.20699	OL638159	OL689845	OL689847	OL689849
*T. siamensis*	CBS 475.88 = IMI 323204	JN899385	JX091379	KF741960	KM023279
*T. soli*	NRRL 62165	MH793074	MH792947	MH793011	MH793138
*T. sparsus*	AS3.16003	MT077182	MT083924	MT083925	MT083926
*T. stellenboschiensis*	CBS 135665 = IBT 32631	JX091471	JX091605	JX140683	MN969157
*T. stipitatus*	CBS 375.48 = NRRL 1006	JN899348	KM111288	KF741957	KM023280
*T. stollii*	CBS 408.93	JX315674	JX315633	JX315646	JX315712
*T. striatoconidium*	CBS 550.89 = DTO418-H4	MN431418	MN969441	MN969360	MT156347
*T. thailandensis*	CBS 133147 = KUFC 3399	JX898041	JX494294	KF741940	KM023307
*T. tumuli*	NRRL 6013	MH793071	MH792944	MH793008	MH793135
*T. veerkampii*	CBS 500.78 = IBT 14845	KF741984	KF741918	KF741961	KX961279
*T. verruculosus*	NRRL 1050 = CBS 388.48	KF741994	KF741928	KF741974	KM023306
*T. versatilis*	IMI 134755 = CBS 140377	KC962111	KC992270	MN969319	MN969161
*T. virens*	CGMCC 3.25207	ON563152	ON231297	ON470840	ON470841
*T. viridis*	CBS 114.72 = NRRL 5575	AF285782	JX494310	KF741935	JN121430
*T. viridulus*	CBS 252.87 = FRR 1863	JN899314	JX091385	KF741943	JF417422
*T. wushanicus*	CGMCC 3.20481	MZ356356	MZ361347	MZ361354	MZ361361
*T. xishaensis*	CGMCC 3.17995	KU644580	KU644581	KU644582	MZ361364
*T. yunnanensis*	KUMCC 18-0208	MT152339	MT161683	MT178251	ON703690 (JGT1-103)*
*T. zhenhaiensis*	AS3.16102	MZ045697	MZ054636	MZ054639	MZ054633
***T. disparis* sp. nov.**	**AS3.26221** ^**T**^	**PP544888**	**PP566271**	**PP566276**	**PP555175**
***T. funiformis* sp. nov.**	**AS3.26220** ^**T**^	**PP544886**	**PP566269**	**PP566274**	**PP555173**
	**AS3.26225**	**PP544887**	**PP566270**	**PP566275**	**PP555174**
***T. jianfengicus* sp. nov.**	**AS3.26253** ^**T**^	**PP544889**	**PP566272**	**PP566277**	**PP555176**
	**JFL34-5**	**PP544890**	**PP566273**	**PP566278**	**PP555177**
*T. assiutensis* (outgroup)	CBS 147.78	JN899323	KJ865720	KJ885260_	KM023305

**Note:**

* The sequences of the ex-types are unavailable in GenBank, those of other strains are used instead. The proposed new species and their strains as well as the GenBank numbers of four genetic markers are in boldface.

In a survey of *Talaromyces* species in China, we discovered five distinctive *Talaromyces* strains and propose here three new species represented by them belonging to sect. *Talaromyces*, namely, *T. disparis* sp. nov., *T. funiformis* sp. nov. and *T. jianfengicus* sp. nov.

## Materials and Methods

### Isolation of fungi

*Talaromyces* strains were isolated from soil samples collected from Beijing, Hainan Province and Hebei Province using the method described by Malloch (1981), with dichloran rose Bengal chloramphenicol agar (DRBC) as the isolation medium. Five distinctive *Talaromyces* strains were isolated and deposited in China General Microbiological Culture Collection (CGMCC) as AS3.26221 (BWL1-2L), AS3.26224 (JXL1-2), AS3.26225 (SJZ2-4), AS3.26220 (BWL1-2), and AS3.26253 (JFL18-1), and one strain, *i.e*., AS3.26224 (JXL1-2) was identified as *T. qii*.

### Morphological observation

For examination of macro-morphological characters, the culturing media of Czapek agar (Cz), Czapek yeast autolysate agar (CYA), 5% malt extract agar (MEA), yeast extract sucrose agar (YES) and Oatmeal agar (OA), and the incubation temperature and time were used according to Pitt & Hocking (2009), Samson et al. (2010). Color names of Ridgway (1912) were referenced in describing the colors of conidia *en masse*, mycelium, exudate and/or soluble pigment. For examination of microscopic characters, the procedure described by Xu et al. (2022) was followed.

### Phylogenetic analysis

Isolation of genomic DNA was carried out in accordance to the procedure of Scott et al. (2000). Partial *BenA*, *CaM*, *Rpb2* and ITS sequences were amplified with primers described by Glass & Donaldson (1995), Wang (2012), Jiang et al. (2018) and White et al. (1990), respectively. PCR reagent mixture formulation and reaction parameters were referenced to Zang et al. (2023). Amplicons of the target loci were purified and sequenced, then proofread according to the method described by Xu et al. (2022). The finished sequences without primer sequences were deposited in GenBank (**BWL1-2 = AS3.26220**: ITS = PP544886, *BenA* = PP566269, *CaM* = PP566274, *Rpb2* = PP555173; **BWL1-2L = AS3.26221**: ITS = PP544888, *BenA* = PP566271, *CaM* = PP566276, *Rpb2* = PP555175; **JFL18-1 = AS3.26253**: ITS = PP544889, *BenA* = PP566272, *CaM* = PP566277, *Rpb2* = PP555176; **JFL34-5**: ITS = PP544890, *BenA* = PP566273, *CaM* = PP566278, *Rpb2* = PP555177; **SJZ2-4 = AS3.26225**: ITS = PP544887, *BenA* = PP566270, *CaM* = PP566275, *Rpb2* = PP555174; **JXL1-2 = AS3.26224**: ITS = MZ220767, *BenA* = MZ220770, *CaM* = MZ220773, *Rpb2* = MZ221212).

Among the 90 species in Table 1, 89 species were selected for the phylogenetic analysis of the concatenated *BenA-CaM-Rpb2* sequences except *T. atkinsoniae* whose *CaM* sequence is unavailable in GenBank. Our six strains and one unidentified strain URM 8665, were included in this *BenA-CaM-Rpb2* sequence matrix, thus there were 96 strains in total. Moreover, to confirm the novelty of strains AS3.26253 and JFL34-5, an analysis of *BenA-CaM-*ITS sequences of 101 strains was carried out including the above 96 strains and one additional strain of *T. oumae-annae*, one additional strain of *T. stellenboschiensis* and three additional strains of *T. verruculosus* (Visagie et al., 2015). In addition, individual analyses of *BenA*, *CaM* and ITS sequences of the above 101 strains, and *Rpb2* sequences of the above 96 strains were also conducted. In all the analyses, *T. assiutensis* of sect. *Trachyspermi* was selected as the outgroup species.

Sequence datasets of the concatenated and individual loci were aligned with MUSCLE implemented in MEGA 6 (Tamura et al., 2013), the alignments were checked and edited to generate sequence matrices, then analyzed with the Maximum Likelihood (ML) method, in which the bootstrap method was used to test the phylogeny for running 1,000 replications. The substitution model and rates among sites were determined by the tool “Find Best DNA/Protein Models (ML)” of MEGA 6, “K2+G+I” is suitable for all the sequence matrices, in which gaps were treated as partial deletion as suggested by Hall (2013).

### Nomenclature

The electronic version of this article in Portable Document Format (PDF) will represent a published work according to the International Code of Nomenclature for algae, fungi, and plants, and hence the new names contained in the electronic version are effectively published under that Code from the electronic edition alone. In addition, new names contained in this work have been submitted to MycoBank from where they will be made available to the Global Names Index. The unique MycoBank number can be resolved and the associated information viewed through any standard web browser by appending the MycoBank number contained in this publication to the prefix “http://www.mycobank.org/MycoTaxo.aspx?Link=T&Rec=”. The online version of this work is archived and available from the following digital repositories: PeerJ, PubMed Central, and CLOCKSS.

## Results

### Phylogenetic analyses

**Figure 1:**
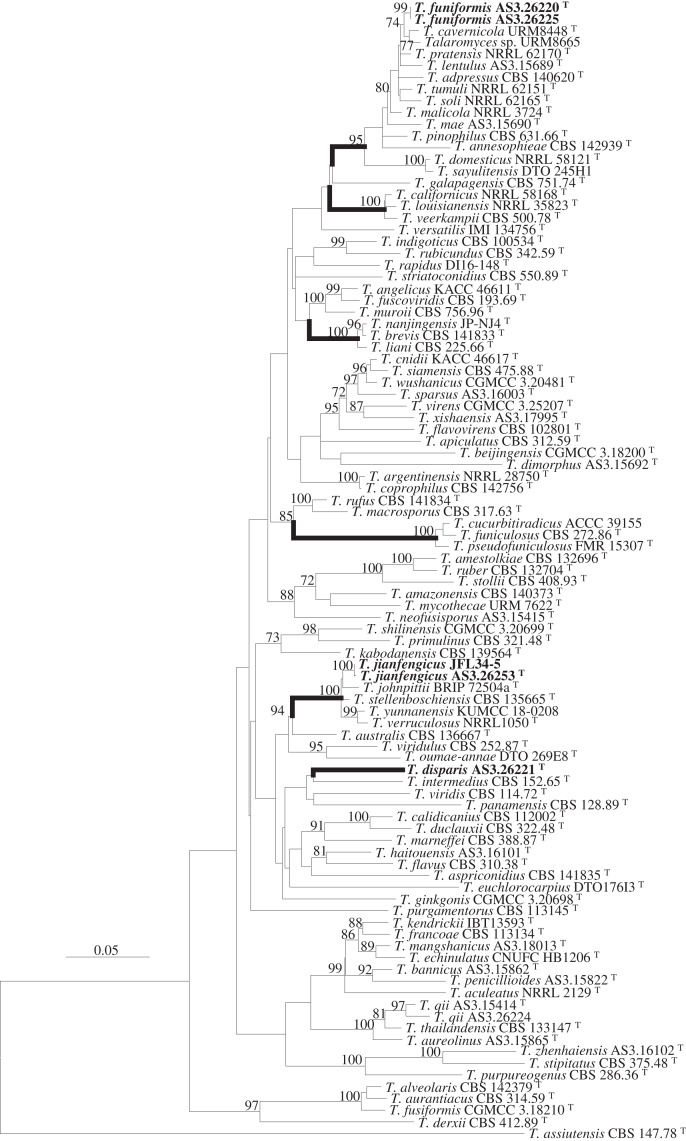
ML phylogram inferred from the concatenated BenA-CaM-Rpb2 sequences showing the three new species in boldface. ML phylogram inferred from the concatenated *BenA-CaM-Rpb2* sequences. Bootstrap percentages over 70% derived from 1,000 replicates are indicated at the nodes. New species are indicated in boldface. Bar = 0.05 substitutions per nucleotide position.

PCR amplification generated ca. 420, 670, 826 and 560 bp amplicons for *BenA*, *CaM*, *Rpb2* and ITS, respectively. Matrices of *BenA*-*CaM*-*Rpb2*, *BenA*-*CaM*-ITS, *BenA*, *CaM*, *Rpb2*, and ITS sequences consist of 1,277, 1,256, 332, 482, 480 and 442 sites with gaps, respectively. The resulted phylograms from the concatenated and the individual matrices all supported the delineation of three novel species (Fig. 1, Figs. S1–S5).

Strain AS3.26221 forms a unique clade with no closely related members in either the phylogams based on the *BenA-CaM-Rpb2* and *BenA-CaM-*ITS (Fig. 1, Fig. S1) or those based on individual sequence matrices (Figs. S2–S5), thus, the name *T. disparis* sp. nov. is proposed for it. Although it is in a clade with *T. intermedius*, *T. viridis*, *T. panamensis* in the *BenA-CaM-Rpb2* phylogenetic tree, and in a clade with *T. panamensis* in the *BenA-CaM-*ITS tree, there is no bootstrap support. In the *Rpb2* phylogram, it is basal to the clade consisting of *T. aculeatus*, *T. echinulatus*, *T. francoae*, *T. bannicus*, *T. kendrickii*, *T. mangshanicus* and *T. penicillioides* (Fig. S4), but there is still no bootstrap support. Strains AS3.26220 and AS3.26225 group in the *T. pinophilus* complex which includes twelve members, *i.e*., *T. adpressus*, *T. annesophieae*, *T. cavernicola*, *T. domesticus*, *T. lentulus*, *T. mae*, *T. malicola*, *T. pratensis*, *T. sayulitensis*, *T. soli*, *T. pinophilus* and *T. tumuli* in all the phylograms except the ITS phylogram (note: *T. domesticus* and *T. sayulitensis* are not in this complex in *BenA-CaM-*ITS and *CaM* phylograms). The two strains in this study form a clade related to *T. cavernicola* and strain URM 8665 with 74% and 71% bootstrap support in *BenA-CaM-Rpb2* and *BenA-CaM-*ITS phylograms, respectively, but no closely related taxa in *BenA*, *CaM* and *Rpb2* phylograms. Therefore, they may represent a new species and are named here as *T. funiformis* sp. nov. (Fig. 1, Figs. S1–S4). In the phylogram inferred from *BenA-CaM-Rpb2* sequence matrix, strains AS3.26253 and JFL34-5 form one clade in the *T. verruculosus* complex, closely related to the four members of this complex, *i.e*., *T. johnpittii*, *T. stellenboschiensis*, *T. verruculosus*, and *T. yunnanensis* with 100% bootstrap support. Moreover, in the phylograms resulted from *BenA-CaM-*ITS, *BenA*, *CaM*, and *Rpb2* sequence matrices with additional *T. stellenboschiensis* and *T. verruculosus* strains, they still form the clade closely related to the above four members with 98%, 97%, 86%, 100% bootstrap support, respectively, so these two strains are considered a new species in this complex and are named here as *T. jianfengicus* sp. nov. In the ITS phylogram, two proposed new taxa, *i.e*., *T. funiformis* and *T. jianfengicus* cannot be discriminated from their close relatives, while *T. disparis* sp. nov. still forms a solitary clade without any relatives (Fig. S5).

**Figure 2:**
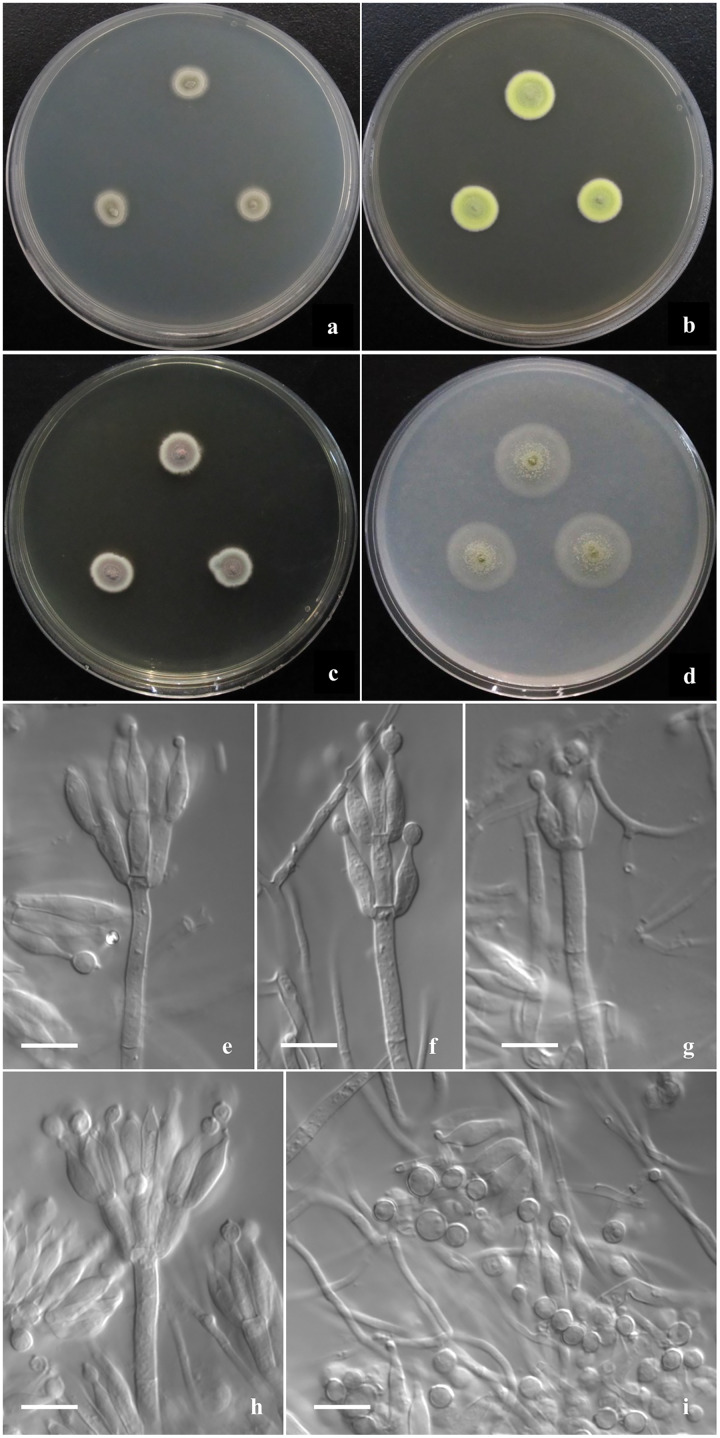
Morphological characters of *T. disparis* sp. nov. Morphological characters of *T. disparis* AS3.26221 ^T^ incubated at 25 °C for 7 days. (A–D) Colonies on CYA, MEA, YES and OA. (E–I) Conidiophores. (I) Conidia. Bar = 10 µm.

**Figure 3:**
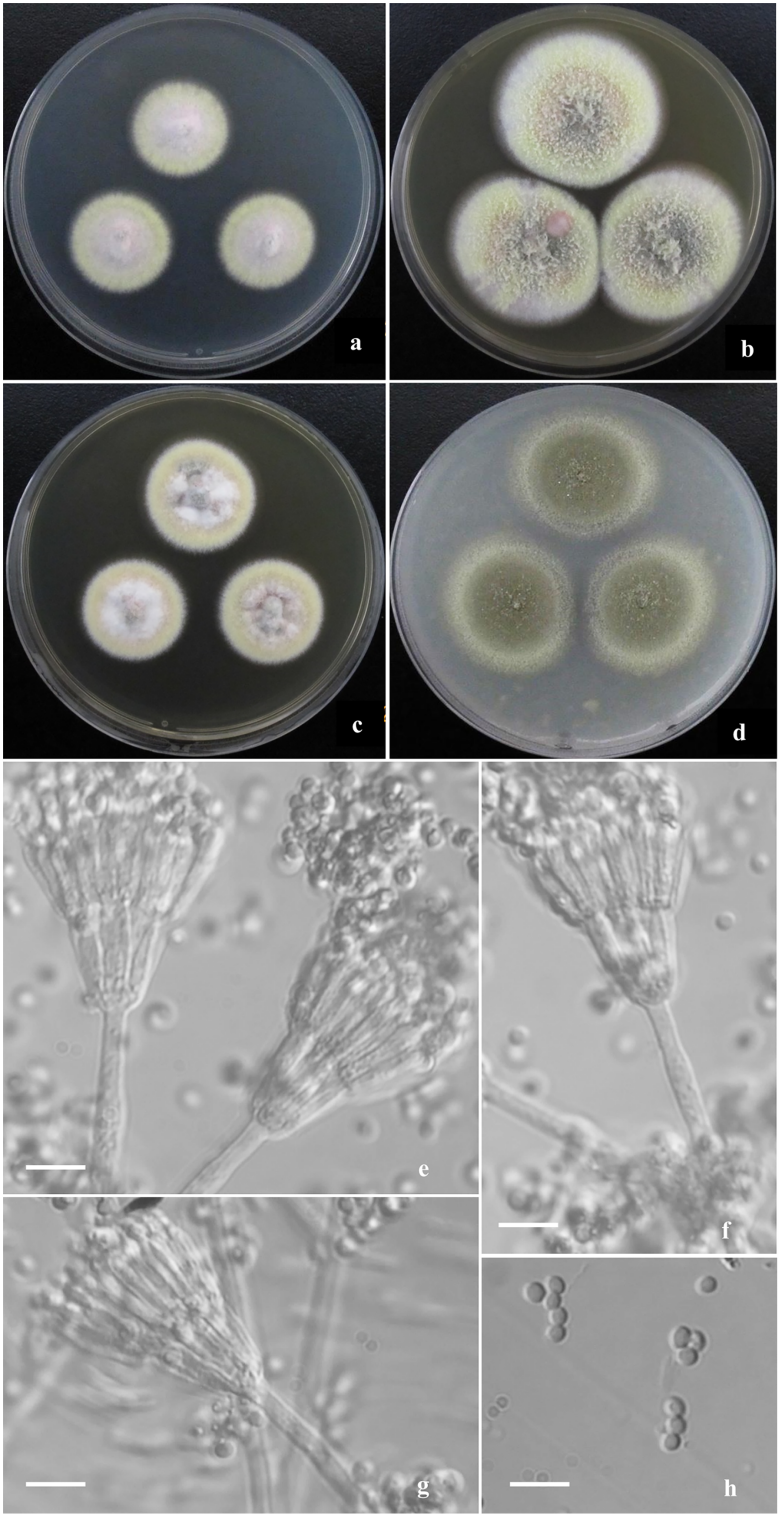
Morphological characters of *T. funiformis* sp. nov. Morphological characters of *T. funiformis* AS3.26220 ^T^ incubated at 25 °C for 7 days. (A–D) Colonies on CYA, MEA, YES and OA. (E–G) Conidiophores. (H) Conidia. Bar = 10 µm.

**Figure 4:**
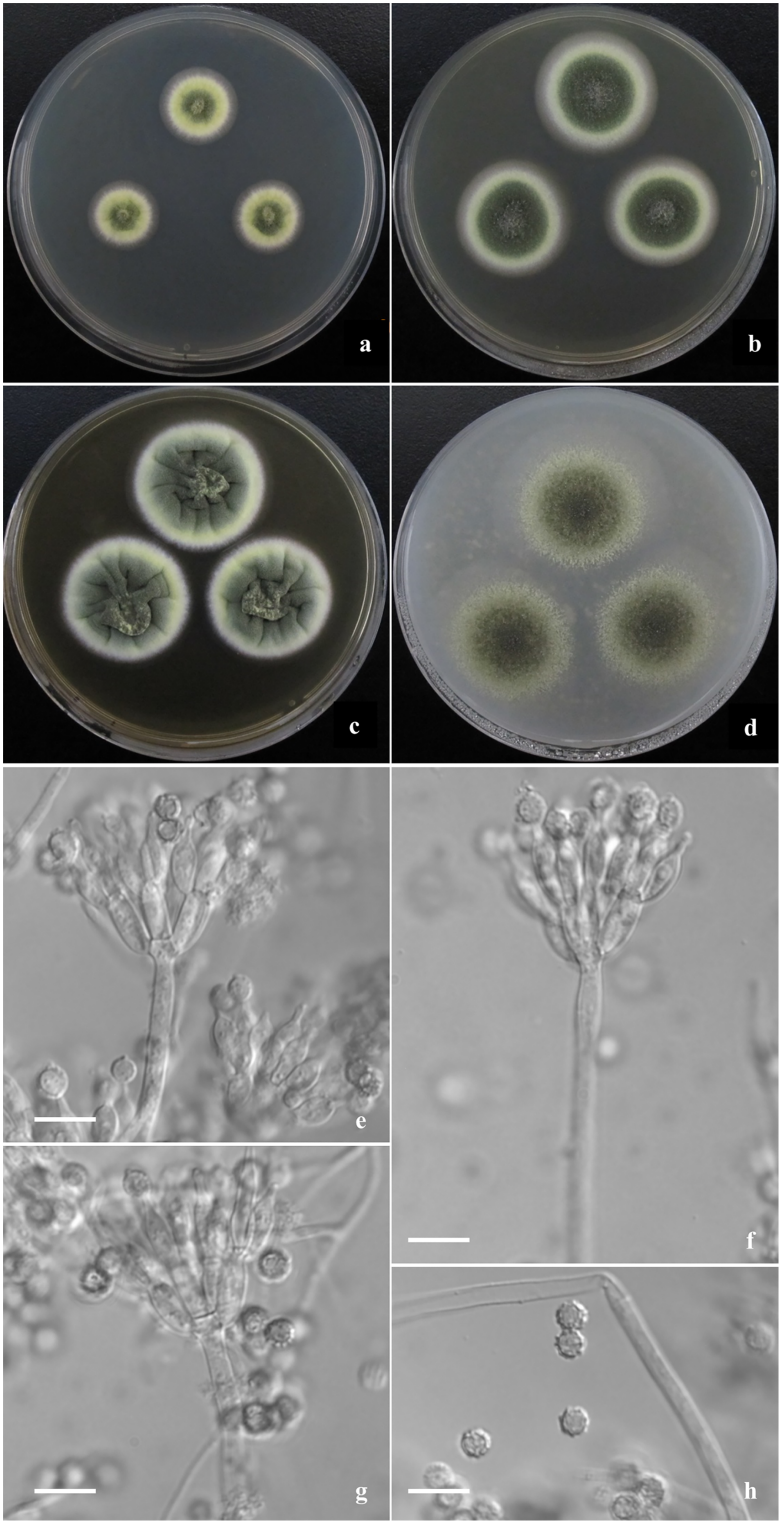
Morphological characters of *T. jianfengicus* sp. nov. Morphological characters of *T. jianfengicus* AS3.26253 ^T^ incubated at 25 °C for 7 days. (A–D) Colonies on CYA, MEA, YES and OA. (E–G) Conidiophores. (H) Conidia. Bar = 10 µm.

**Descriptions of new species (Figs. 2–4)**

***Talaromyces disparis*** Y. Ruan & L. Wang, sp. nov.

MycoBank No: MB 853638

(Fig. 2)

**Etymology**. The specific epithet refers to its penicilli in different patterns and conidia in different shapes and dimensions.

**Holotype**. CHINA. HAINAN: Changjiang, Bawang Ridge Nature Reserve, from soil, 19°7′53"N 109°8′2″E, 1,000 m, April 30, 2019, *W.-C. Wang* BWL1-2L, ex-type culture AS3.26221 (holotype: HMAS 352912, from the dried culture of AS3.26221 on MEA). GenBank: *BenA* = PP566271, *CaM* = PP566276, ITS = PP544888, *Rpb2* = PP555175.

**Diagnosis**. This new taxon is characterized by low growth rate, velvety texture, limited to moderate sporulation; biverticillate, monoverticillate and irregular penicilli, and bearing polymorphic smooth-walled conidia with a portion of abnormally large globose ones.

## Description

Colonies 10–11 mm diam on CYA at 25 °C after 7 d, low, plane, margins fimbriate; texture velvety; sporulation moderate, conidia *en masse* Olive-Buff to Dark Olive-Buff (R. Pl. XL); mycelium white; no exudate and soluble pigment; reverse yellowish white. Colonies 13–14 mm diam on MEA at 25 °C after 7 d, slightly deep, with weak concentric plicates, margins regular; texture velvety; sporulation moderate, conidia *en masse* Light Cress Green (R. Pl. XXXI); mycelium Green-Yellow (R. Pl. V); exudate and soluble pigment absent; reverse Capucine Buff (R. Pl. III). Colonies 12–13 mm diam on YES at 25 °C after 7 d, low, plane; margins fimbriate; texture velvety; sporulation moderate to limited, conidia *en masse* Light Olive-Gray (R. Pl. LI), mycelium Vinaceous-Fawn to Deep Olive-Buff or Dark Olive-Buff (R. Pl. XL), white at margins; no exudate and soluble pigment; reverse Vinaceous-Rufous (R. Pl. XIV). Colonies 18–20 mm diam on OA at 25 °C after 7 d, sparse, low, plane, margins regular; texture velvety; sporulation limited in centres, conidia *en masse* Cress Green (R. Pl. XXXI); mycelium Naphthalene Yellow (R. Pl. XVI) in central areas while white elsewhere; exudate absent or limited, clear; no soluble pigment; reverse yellowish white. Colonies 11–12 mm diam on Cz at 25 °C after 7 d, low, plane, sparse, margins regular; texture velvety; sporulation absent; mycelium Sulphur Yellow (R. Pl. V); no exudate and soluble pigment; reverse yellowish white. On CYA at 37 °C after 7 d, no growth.

Conidiophores rising from surface hyphae; stipes 60–80 (–120) × 3–3.5 μm; penicilli biverticillate, monoverticillate and irregular; metulae (3–) 6–8 per vertical, 10–12 × 3–4 μm; phialides (3–) 4–6 per verticil, ampuliform, 10–12 (–14) × 3–4 μm; conidia ovoid, globose to lemon-shaped, 4–4.5 × 3.5–4 μm, abnormally large globose ones about 5–6 μm, walls thick and smooth, some with connectives at both ends.

***Talaromyces funiformis*** Y. Ruan & L. Wang, sp. nov.

MycoBank No. MB 853639

(Fig. 3)

**Etymology**. The specific epithet refers to the funiculose appearance on CYA, MEA and YES.

**Holotype**. CHINA. HAINAN: Changjiang, Bawang Ridge Nature Reserve, from soil, 19°7′53″N 109°8′2″E, 1,000 m, April 30, 2019, *W.-C. Wang* BWL1-2, ex-type culture AS3.26220 (holotype: HMAS 352911, from the dried culture of AS3.26220 on MEA). GenBank: *BenA* = MZ220770, *CaM* = MZ220773, ITS = MZ220767, *Rpb2* = MZ221212.

**Diagnosis**. This new taxon is characterized by producing mycelial funicles on CYA, MEA and YES, sparse sporulation on Cz, CYA, MEA and YES while abundant on OA, low growth rate at 37 °C; appressed biverticillate penicilli and globose to pyriform conida with smooth to finely rough walls.

## Description

Colonies 26–28 mm diam on CYA at 25 °C after 7 d, low, plane, margins fimbriate; texture velvety and sparsely funiculose and floccose; sporulation sparse, conidia *en masse* light Pea Green (R. Pl. XLVII); mycelium Pale Vinaceous-Fawn (R. Pl. XL) in central areas and Light Greenish Yellow (R. Pl. V) at margins; no exudate and soluble pigment; reverse Xanthine Orange (R. Pl. III) in central areas, fading into Pale Orange-Yellow (R. Pl. III) at marginal areas. Colonies 38–40 mm diam on MEA at 25 °C after 7 d, slightly deep, plane, margins regular; texture velvety and densely funiculose and floccose; sporulation moderate to sparse, conidia *en masse* Pea Green (R. Pl. XLVII); mycelium Light Greenish Yellow (R. Pl. V); exudate and soluble pigment absent; reverse Mars Orange to Orange Rufous (R. Pl. II). Colonies 30–32 mm diam on YES at 25 °C after 7 d, low, irregularly sulcate; margins fimbriate; texture velvety and sparsely funiculose and floccose; sporulation sparse, conidia *en masse* Olive-Gray (R. Pl. LI); mycelium Pale Salmon Color (R. Pl. XIV) in central areas while Pale Yellow-Orange (R. Pl. III) at margins; exudate limited, brown; no soluble pigment; reverse Xanthine Orange (R. Pl. III), fading into Pale Orange Yellow (R. Pl. III) at marginal areas. Colonies 35–37 mm diam on OA at 25 °C after 7 d, low, plane, margins fimbriate; texture velvety; sporulation abundant, conidia *en masse* Olive-Gray (R. Pl. LI); mycelium pale yellow; exudate absent or limited, clear; no soluble pigment; reverse Cinnamon Rufous (R. Pl. XIV), fading into light yellow at marginal areas. Colonies 12–14 mm diam on Cz at 25 °C after 7 d, low, plane, margins fimbriate; texture velvety; sporulation sparse, limited in centres, conidia *en masse* Deep Grayish Olive (R. Pl. XLVI); mycelium white mingled with Pale Flesh Color (R. Pl. XIV); no exudate and soluble pigment; reverse Salmon Buff (R. Pl. XIV). Colonies 10–12 mm diam on CYA at 37 °C, deep, margins regular; texture densely floccose; no sporulation; mycelium white; no exudate and soluble pigment; reverse Salmon Buff to Cinnamon-Buff (R. Pl. XIV).

Conidiophores rising from mycelial funicles; stipes 150–300 × 2.5–3.5 μm, smooth-walled; penicilli biverticillate, appressed; metulae 6–8 per vertical, 10–13 × 2–2.5 μm; phialides 6–8 per verticil, acerose, 10–12 × 2–2.5 μm; conidia globose to pyriform, 2–2.5 μm, walls smooth to finely rough.

Additional strains: CHINA, HEIBEI: Shijiazhuang, Daguo Village, from farm soil, 38°5′9″N 114°25′18″E, 70 m, August 20, 2020, *S.-Z. Wei* SJZ2-4 = AS3.26225. GenBank: *BenA* = PP566270, *CaM* = PP566275, ITS = PP544887, *Rpb2* = PP555174.

***Talaromyces jianfengicus*** Y. Ruan & L. Wang, sp. nov.

MycoBank No. MB 853553

(Fig. 4)

**Etymology**. The specific epithet refers to the locale where the ex-type strain was isolated.

**Holotype**. CHINA: HAINAN: Ledong, Jianfeng Ridge Nature Reserve, from soil, 18°42′14″N 108°49′33″E, 600 m, April 21, 2019, *W.-C. Wang* JFJ18-1, ex-type culture AS3.26253 (holotype: HMAS 352913, from the dried culture on MEA). GenBank: *BenA* = PP566272, *CaM* = PP566277, ITS = PP544889, *Rpb2* = PP555176.

**Diagnosis**. This new taxon is characterized by velvety colony texture with moderate to abundant elm-green conidia *en masse* and green-yellow mycelium, biverticillate penicilli, ampuliform phialides and globose conidia with verrucose walls.

## Description

Colonies 18–19 mm diam on CYA at 25 °C after 7 d, low, plane, margins fimbriate; texture velvety; sporulation moderate, conidia *en masse* Light Elm Green (R. Pl. XVII); mycelium Chalcedony Yellow (R. Pl. XVII), white at margins; no exudate and soluble pigment; reverse Pale Orange-Yellow (R. Pl. III). Colonies 29–31 mm diam on MEA at 25 °C after 7 d, low, plane, margins regular; texture velvety; sporulation abundant, conidia *en masse* Elm Green (R. Pl. XVII); mycelium Chalcedony Yellow (R. Pl. XVII), white at margins; no exudate and soluble pigment; reverse Warm buff (R. Pl. XV). Colonies 34–35 mm diam on YES at 25 °C after 7 d, low, convolute centrally, irregularly sulcate densely; margins regular; texture velvety; sporulation abundant, conidia *en masse* Yew Green (R. Pl. XXXI); mycelium Pale Greenish Yellow R. Pl. V), white at margins; no exudate and soluble pigment; reverse Ochraceous-Salmon to Warm Buff (R. Pl. XV). Colonies 38–40 mm diam on OA at 25 °C after 7 d, low, plane, margins wide, regular; texture velvety; sporulation abundant, conidia *en masse* Cerro Green (R. Pl. V); mycelium Pale Viridine Yellow (R. Pl. V), white at margins; exudate limited, clear; no soluble pigment; reverse Chalcedony Yellow (R. Pl. XVII). Colonies 16–18 mm diam on Cz at 25 °C after 7 d, low, plane, margins wide, regular; texture velvety; sporulation limited to moderate, conidia *en masse* Elm Green (R. Pl. XVII); mycelium Chalcedony Yellow (R. Pl. XVII), white at margins; no exudate and soluble pigment; reverse Capucine Buff (R. Pl. III). Colonies 6–7 mm diam on CYA at 37 °C, deep, margins regular; texture velvety; no sporulation; mycelium white; no exudate and soluble pigment; reverse Pinkish Cinnamon (R. Pl. XXIX).

Conidiophores rising from surface and aerial hyphae; stipes 150–200 × 2.5–3 μm when from surface hyphae, and 10–50 μm long when from aerial hyphae, smooth-walled; penicilli biverticillate; metulae 8–10 per stipe, 8–10 × 3.5–4 μm; phialides 6–8 per metula, ampuliform, 10–12 × 3.5–4 μm; conidia globose, 4–5 μm, walls verrucose.

Additional strains: CHINA: HAINAN: Ledong, Jiangfeng Ridge Nature Reserve, from soil, 18°42′14″N 108°49′33″E, 600 m, April 21, 2019, *W.-C. Wang* JFJ34-5, GenBank: *BenA* = PP566273, *CaM* = PP566278, ITS = PP544889, *Rpb2* = PP555176.

## Discussion

Sect. *Talaromyces* is the largest section in *Talaromyces*, including species that commonly grow fast, produce spreading velvelty to floccose colonies more than 30 mm diam. on MEA, and their penicilli are typically biverticillate, though some produce a portion of monoverticillate, terverticillate (with subterminal branches) or irregular penicilli. Some members of this section grow slowly, *e.g*., *T. bannicus*, *T. mangshanicus* and *T. marneffei*, or form colonies with synnemata or funicles, such as, *T. calidicanius*, *T. duclauxii*, *T. funiculosus*, *T. pinophilus*, *T. pseudostromaticus*, but biverticillate penicilli are usually produced, irrespective of whether their phialides are acerose or ampuliform. Phylogenetically, species in this section are usually distantly related, but some closely related taxa compose species complexes, for instance, *T. pinophilus* complex includes twelve members, *i.e*., *T. cavernicola*, *T. pratensis*, *T. lentulus*, *T. adpressus*, *T. tumuli*, *T. soli*, *T. malicola*, *T. pinophilus*, *T. mae*, *T. domesticus*, *T. sayulitensis*, *T. annesophieae*; *T. verruculosus* complex contains four members, namely, *T. johnpittii*, *T. stellenboschiensis*, *T. yunnanensis*, *T. verruculosus*; *T. liani* complex consists of *T. nanjingensis*, *T. liani*, *T. brevis*; *T. veerkampii* complex is comprised of *T. californicus*, *T. louisianensis*, *T. veerkampii*; and *T. funiculosus* complex comprises *T. cucurbitiradicus*, *T. funiculosus, T. pseudofuniculosus* (*e.g*., Fig. 1). Members of these species complexes are not easily discriminated from each other by using morphology, especially micro-morphology.

Using the polyphasic taxonomy that integrates morphological and phylogenetic characters, we established a new species based on one single strain, since it is phylogenetically distinctive and its morphological characters are obviously distinguishable from other species. *T. disparis* is a unique taxon that has no close relatives in *Talaromyces* based on the phylogenetic analysis (Fig. 1, Figs. S1–S5). Though it is in a clade with *T. intermedius*, *T. viridis* and *T. panamensis* in *BenA-CaM-Rpb2* phylogram, no bootstrap support is presented. In morphological characters, it is distinctive in low growth rate at 25 °C (CYA: 10–11 mm, MEA: 13–14 mm, YES: 12–13 mm, Cz: 11–12 mm), produces polymorphic conidiophores with biverticillate, monoverticillate and irregular penicilli, bears polymorphic conidia that are ovoid, globose to lemon-shaped commonly measured 4–4.5 × 3.5–4 μm, while with abnormally large globose ones about 5–6 μm. There are few species in sect. *Talaromyces* that grow slowly on conventional culturing media at 25 °C, such as *T. bannicus*, *T. mangshanicus* and *T. viridis* (Yilmaz et al., 2014; Wang et al., 2017; Wei, Xu & Wang, 2021). *T. viridis* is readily distinguished from *T. disparis* by producing ascomata and abnormal anamorphs with solitary phialides and fusiform to ellipsoidal conidia. Besides the restricted growth, *T. bannicus* and *T. mangshanicus* both produce biverticillate, monoverticillate and irregular conidiophores with ampuliform phialides similar to those of *T. disparis*. The definitive difference between *T. disparis* and *T. mangshanicus* is that the conidia of the latter are subglobose to ellipsoidal with echinulate walls, obviously different from those of the new species, which are polymorphic with smooth walls. *T. bannicus* also bears polymorphic conidia with abnormally large ones similar to those of the new species, but their shapes are commonly pyriform to ellipsoidal with walls conspicuously echinulate to verrucose. In all the phylograms, the new species is distantly separated from *T. bannicus* and *T. mangshanicus* (Fig. 1, Figs. S1–S5).

*Talaromyces funiformis* belongs to the *T. pinophilus* complex. There are now thirteen members in this species complex, which commonly show floccose and funiculose colony appearance, grow well at 37 °C (except *T. annesophieae* which does not grow), and produce compact penicilli bearing acerose phialides and globose, subglobose to broadly ellipsoidal conidia with smooth to finely rough walls (Visagie et al., 2014; Crous et al., 2017; Jiang et al., 2018; Peterson & Jurjević, 2019; Alves et al., 2022). In the phylograms based on *BenA-CaM-Rpb2* and *BenA-CaM-*ITS, *T. funiformis* is weakly related to *T. cavernicola* and the unidentified strain URM8665 with 74% and 71% bootstrap support, respectively. But according to the analyses based on individual loci, except that the ITS region is unable to distinguish between *T. funiformis* and *T. cavernicola*, the *BenA*, *CaM*, and *Rpb2* phylograms all show that they are unrelated. Also morphologically, the floccose and funiculose colony texture, light greenish yellow mycelium and sparse sporulation on CYA, MEA, YES and Cz show similarity to *T. cavernicola*, but *T. funiformis* can be discriminated from *T. cavernicola* by growing more slowly on CYA, YES and Cz at 25 °C (CYA: 26–28 mm *vs*. 31–35 mm, YES: 30–32 mm *vs*. 40–47 mm, Cz: 12–14 mm *vs*. 18–19 mm) and on CYA at 37 °C (10–12 mm *vs*. 34–38 mm), while much faster on OA at 25 °C (35–37 mm *vs*. 24–28 mm diam). Moreover, it shows velvety colony texture and abundant sporulation on OA, where *T. cavernicola* shows floccose texture with poor sporulation. The low growth rate at 37 °C on CYA (10–12 mm) and velvety texture with abundant sporulation on OA of *T. funiformis* can be used to distinguish it from the other members in this complex, which commonly grow moderately to fast at 37 °C and show floccose and funiculose texture on OA, *e.g*., *T. pinophilus* (24–40 mm), *T. domesticus* (30–38 mm), *T. pratensis* (25–30 mm), *T. soli* (23–28 mm), *T. tumuli* (21–35 mm), *T. adpressus* (35–38 mm), *T. sayulitensis* (32–40 mm), *T. lentulus* (18–21 mm), *T. mae* (17–18 mm). The only exception is *T. malicola* which grows slowly at 37 °C on CYA (9–10 mm) similar to *T. funiformis* (10–12 mm), but the floccose and funiculose texture on OA and low growth rate on MEA (29–31 mm *vs*. 38–40 mm) of *T. malicola* still can be relied on to distinguish it from the new species.

The last new species, *T. jianfengicus* belongs to *T. verruculosus* complex including *T. johnpittii*, *T. stellenboschiensis*, *T. yunnanensis* and *T. verruculosus*. These members commonly grow moderately to fast on MEA, YES and OA (note: uncertain in *T. johnpittii* whose data are unavailable), produce green-yellow mycelium on CYA, MEA and YES, and moderate to abundant conidia in dark green (elm green) and color *en masse* on MEA, their penicilli usually broadly biverticillate with amuliform phialides bearing globose conidia with echinulate to verrucose walls. The new species produces similar colony appearance, and conidiophores and conidia in shape and dimension to those of *T. stellenboschiensis*, *T. yunnanensis* and *T. verruculosus*, but it still can be discriminated from them by much slow growth at 25 °C on CYA (18–19 mm *vs*. 40–45 mm, 35–37 mm, 32–35 mm, respectively) and MEA (29–31 mm *vs*. 40–42 mm, 50–53 mm, 35–36 mm, respectively), and further distinguished from *T. stellenboschiensis* and *T. verruculosus* by restricted growth at 37 °C on CYA (6–7 mm *vs*. 35–40 mm, 25–26 mm, respectively). There are no growth data on CYA, MEA, YES and OA for *T. johnpittii*, but the new species and *T. johnpittii* are obviously different in penicilli, for example, the new species produces more numerous metulae and phialides than *T. johnpittii* (metulae: 8–10 *vs*. 2–5, phialides: 6–8 *vs*. 2–5), and the phialide collulae of *T. johnpittii* are distinctive in showing periclinal thickened collarettes, while those of the new species are conoidal, which is conventional in *Talaromyces* (Yilmaz et al., 2014; Visagie et al., 2015; Doilom et al., 2020; Lacey et al., 2023).

It is noteworthy that some species in sect. *Talaromyces* are opportunistic pathogens for humans and animals. *T. marneffei* is the only thermally dimorphic species in *Talaromyces*, causing invasive talaromycosis in immunocompromised patients. It is believed that the infection begins with the inhalation of conidia. However, it is puzzling how this fungus produces enough conidia to reach a threshold density for infection in natural habitats, such as soil, since the growth of *T. marneffei* on artificial culturing media is very slow and the sporulation is sparse. After inhaled, the infectious conidia are primarily engulfed by alveolar macrophages where they germinate to form yeast cells due to 37 °C and the intracellular environment of macrophages. These yeast cells reprogram metabolism pathways to adapt nutrient limitation and synthesize various kinds of virulence factors to survive the stress in macrophages, for example, heat shock protein (HSP), catalase-peroxidase, superoxide dismutase (SOD), melanin and mannoproteins, *etc*. Some of these proteins, such as HSP, melanin and mannoproteins are included in extracellular vesicles (EVs) secreted into the cytoplasm of macrophage, which stimulate macrophages to produce various inflammatory factors with antimicrobial activity. On the other hand, macrophages may shelter the pathogen from the fungicidal activity of neutrophils (Ellett et al., 2018; Yang et al., 2021; Pruksaphon et al., 2022, 2023; Wang, Han & Chen, 2023). Thus, the pathogenic mechanism of *T. marneffei* still remains not fully understood.

Many other species in this section were reported to cause talaromycosis, for instance, *T. alveolaris*, *T. amestolkiae*, *T. aurantiacus*, *T. cnidii*, *T. funiculosus, T. indigoticus*, *T. kabodanensis*, *T. pinophilus*, *T. purpureogenus*, *T. rapidus*, *T. ruber*, *T. stipitatus*, and *T. stollii* (Yilmaz et al., 2014; Guevara-Suarez et al., 2016, 2017; Guo et al., 2021; Bacon et al., 2022; Sharma & Nonzom, 2022), whereas, some of these species may be misidentified or in doubt. For example, isolate DI16-138 was identified as “*T. cnidii*” by Guevara-Suarez et al. (2016), but using its *BenA* sequence (LT559077) as the query to BLASTn with “sequences from type material”, we found no *T. cnidii* sequences in the result, and there are nine nucleotides different from that of the ex-type strain of *T. cnidii* KACC 46617 (KF183641). Guo et al. (2021) identified an isolate PUMCH_Q056 from clinical specimens as “*T. aurantiacus*”, but the *BenA* (MW148866) and *Rpb2* (MW122767) sequences of the isolate cannot confirm the identification, in addition, according to Bacon et al. (2022), “*T. aurantiacus*” was isolated from the central nervous system of a Labrador retriever but the ITS sequence (MZ338025) represents *Penicillium canescens* in GenBank. Sharma & Nonzom (2022) reported *T. stipitatus* causing superficial mycosis in India, but based on the ITS sequence (MT994164) of their isolate BHS I, it is supposed to be *T. zhenhaiensis* (ITS= MZ045697).

Irrespective of those pathogenic members, many species in sect. *Talaromyces* are potent lignocellulolytic enzyme producers and some species have played an important role in the research and application of degrading vegetal biomass that contains cellulose, hemicelluloses, lignin and pectin. Though *Trichoderma reesei* has been the dominant fungus in studies and industries of ignocellulolytic enzymes, its genome harbors fewer genes encoding cellulase, hemicellulase, especially β-glucosidase than certain *Talaromyces* species do (Martinez et al., 2008). For example, as the first *Talaromyces* species utilized in degrading lignocellulosic biomass, *T. pinophilus* (as “*T. cellulolyticus*”) presents higher glucan-hydrolyzing and xylan-hydrolyzing activity in its cellulase mixture than *Trichoderma reesei* does (Fujii et al., 2009), and the genome of *T. pinophilus* has much more cellulolytic and hemicellulolytic enzyme genes compared with other cellulase-producing fungi (Li et al., 2017). *T. funiculosus* is also proved to be a good producer for cellulolytic and hemicellulolytic enzymes with more β-glucosidases than *Trichoderma reesei* produces (Ogunmolu et al., 2015; Pasari et al., 2023). Studies of Goyari et al. (2015) shows that *T. verrucullosus* secretes efficient β-glucosidase, endoglucanase and cellobiohydrolase on natural cellulose substances with continuous activity without end product inhibition due to sufficient β-glucosidase. Studies of de Eugenio et al. (2017) and Prieto et al. (2021) showed that the genome of *T. amestolkiae* encodes more cellulolytic and hemicellulolytic enzymes than that of *Trichoderma reesei*, and the endoxylanase, β-xylosidase, particularly β-glucosidase produced by *T. amestolkiae* would be good candidates for lignocellulolytic enzyme cocktails. In industries, for example, the endo-1, 3(4)-beta-glucanase and endo-1, 4-beta-xylanase from *T. versatilis* have been the feed additive for many kinds of domestic animals (*e.g*., Llanos et al., 2019). All these facts indicate that species in sect. *Talaromyces* are promising candidates in enzyme industries for plant biomass degradation.

Besides producing lignocellulolytic enzymes, species of sect. *Talaromyces* are robust producers of secondary metabolites, such as terpenoids, steroids, alkaloids, polyketides, *etc*. Compared with other sections in *Talaromyces*, species of this section account for the vast majority in producing various secondary metabolites. According to Nicoletti, Bellavita & Falanga (2023), eleven species from marine environment, namely, *T. aculeatus*, *T. amestolkiae*, *T. flavus*, *T. funiculosus*, *T. mangshanicus*, *T. pinophilus*, *T. purpureogenus*, *T. stipitatus*, *T. stollii*, *T. verruculosus*, *T. versatilis*, are the popular producers for new secondary metabolites as well as those shared by other organisms, a large portion of these compounds have antibacterial, anti-proliferative, anti-inflammatory and antioxidant activities, but many aspects of their biotechnological implications remain unexplored.

## Conclusions

*Talaromyces* species are valuable fungal resource either in research work or industrial application, and the species diversity of this genus still needs much investigation. Though the application of environmental metabarcoding technique is widely used in studies on fungal diversity, isolation of pure cultures, discovering and preserving new species are still the prerequisite for in-depth research and exploitation of these biological resources. In this study, we used the serial dilution method in the isolation of fungi and a polyphasic taxonomic approach integrating the morphological and molecular phylogenetic methods in the discovery and proposal of three *Talaromyces* new species. As the third-generation sequencing technology developed, numerous fungal genomes have been sequenced and assembled in high quality, but much expert bioinformatic analysis still needs to be conducted, which may provide clues for understanding how these fungi survive in natural habitats, pathways of producing lignocellulolytic enzymes and secondary metabolites, as well as the pathogenicity of those opportunistic pathogens.

## Supplemental Information

10.7717/peerj.18253/supp-1 Supplemental Information 1 The sequence matrices analyzed in this study.

10.7717/peerj.18253/supp-2 Supplemental Information 2 ML phylogram inferred from the concatenated *BenA-CaM-*ITS sequences showing the three new species in boldface. Bootstrap percentages over 70% derived from 1000 replicates are indicated at the nodes. New species are indicated in boldface. Bar = 0.05 substitutions per nucleotide position.

10.7717/peerj.18253/supp-3 Supplemental Information 3 ML phylogram inferred from partial BenA sequences showing the three new species in boldface. Bootstrap percentages over 70% derived from 1000 replicates are indicated at the nodes. New species are indicated in boldface. Bar = 0.05 substitutions per nucleotide position.

10.7717/peerj.18253/supp-4 Supplemental Information 4 ML phylogram inferred from partial *CaM* sequences showing the three new species in boldface. Bootstrap percentages over 70% derived from 1000 replicates are indicated at the nodes. New species are indicated in boldface. Bar = 0.05 substitutions per nucleotide position.

10.7717/peerj.18253/supp-5 Supplemental Information 5 ML phylogram inferred from partial *Rpb2* sequences showing the three new species in boldface. Bootstrap percentages over 70% derived from 1000 replicates are indicated at the nodes. New species are indicated in boldface. Bar = 0.05 substitutions per nucleotide position.

10.7717/peerj.18253/supp-6 Supplemental Information 6 ML phylogram inferred from partial ITS sequences showing the three new species in boldface. Bootstrap percentages over 70% derived from 1000 replicates are indicated at the nodes. New species are indicated in boldface. Bar = 0.02 substitutions per nucleotide position.

## Additional Information and Declarations

### Data Availability

The following information was supplied regarding data availability: The raw data is available in the Supplemental File.

### New Species Registration

The following information was supplied regarding the registration of a newly described species: MycoBank: http://www.mycobank.org/mb/853638. http://www.mycobank.org/mb/853639. http://www.mycobank.org/mb/853553.
