# Supplementary figures and images for: Three new species of Talaromyces sect. Talaromyces discovered in China
Source: PeerJ. 2024 Oct 11;12:e18253. doi: 10.7717/peerj.18253 (PMC11472791; doi:10.7717/peerj.18253)

Fig. S1. *BenA*-*CaM*-ITS  
partial del

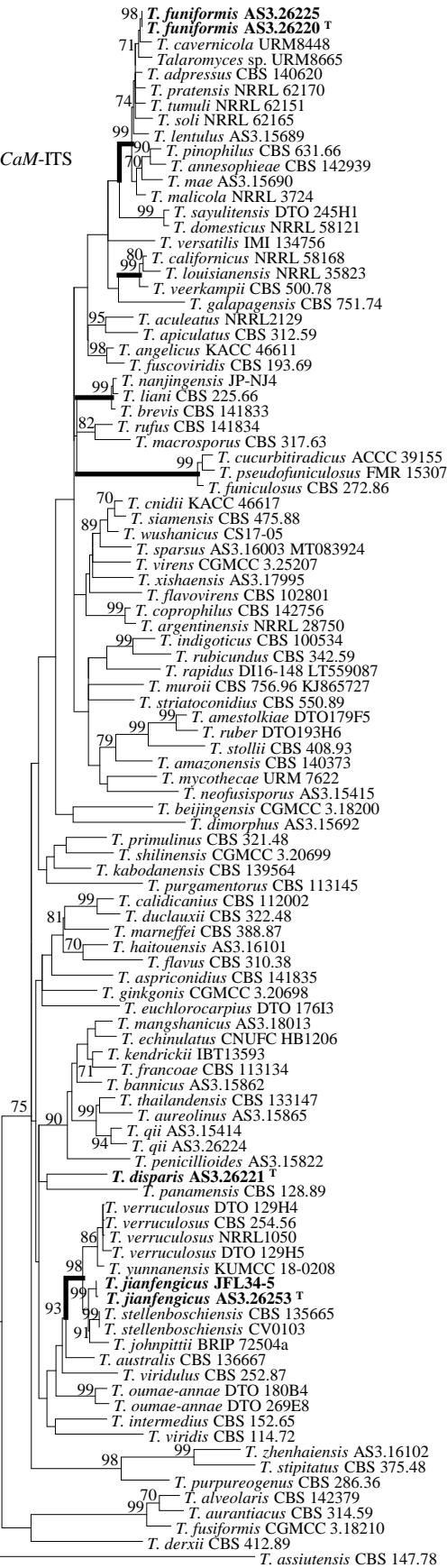

Supplement: Supplemental Information 2 — Bootstrap percentages over 70% derived from 1000 replicates are indicated at the nodes. New species are indicated in boldface. Bar = 0.05 substitutions per nucleotide position. [file peerj-12-18253-s002.pdf]

Fig. S2. *BenA*

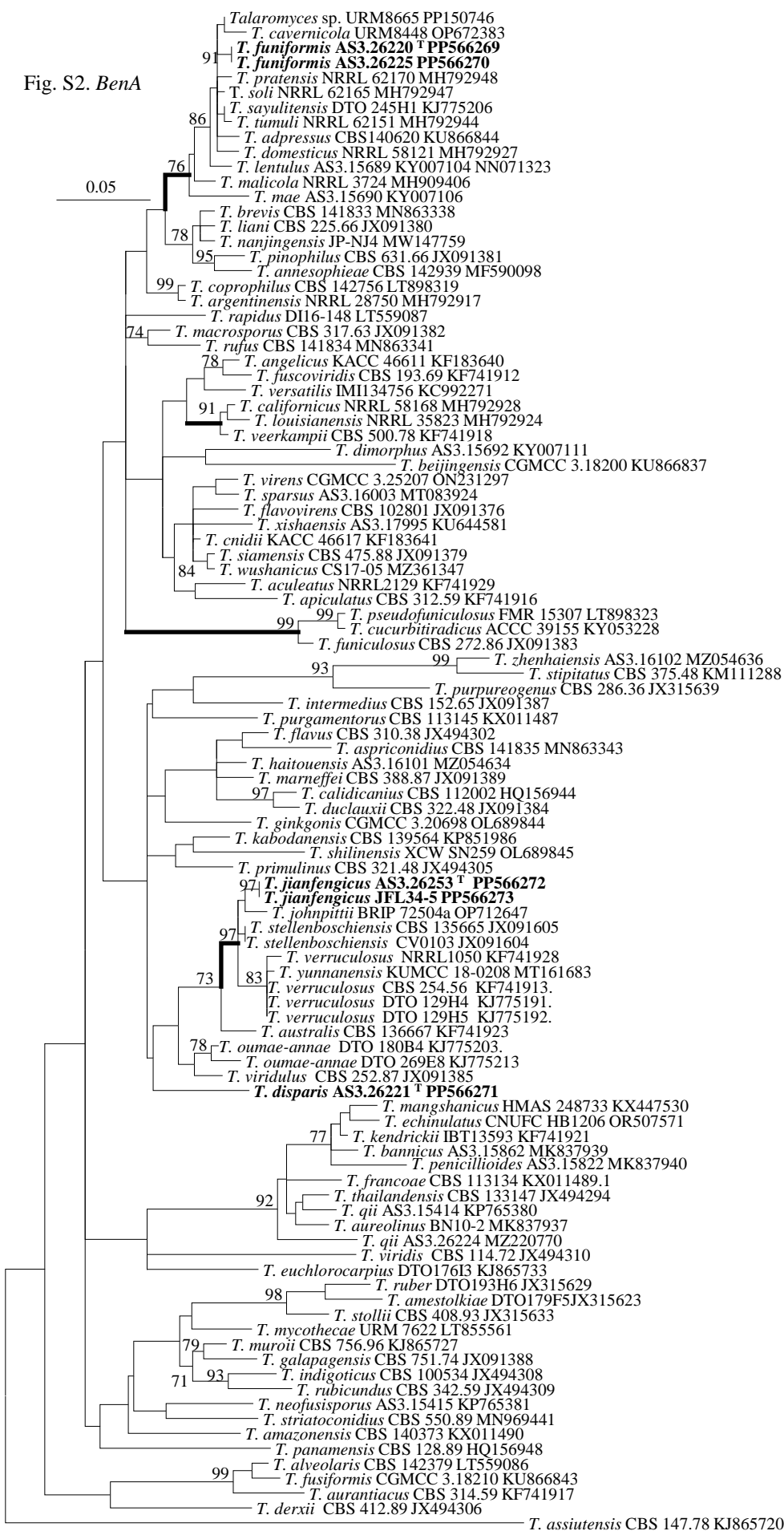

Supplement: Supplemental Information 3 — Bootstrap percentages over 70% derived from 1000 replicates are indicated at the nodes. New species are indicated in boldface. Bar = 0.05 substitutions per nucleotide position. [file peerj-12-18253-s003.pdf]

Fig. S3. *CaM*

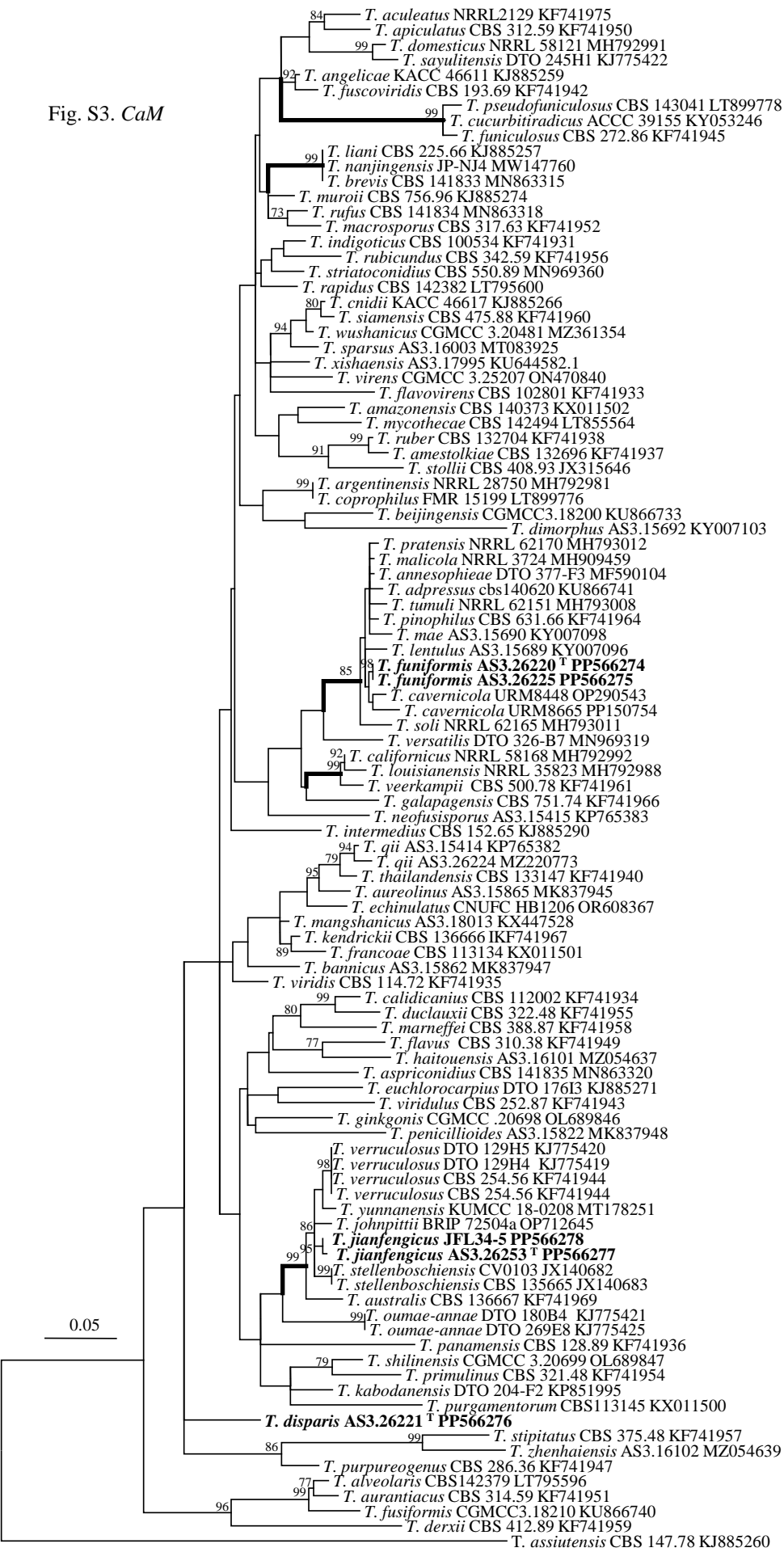

Supplement: Supplemental Information 4 — Bootstrap percentages over 70% derived from 1000 replicates are indicated at the nodes. New species are indicated in boldface. Bar = 0.05 substitutions per nucleotide position. [file peerj-12-18253-s004.pdf]

Fig. S4. *Rpb2*

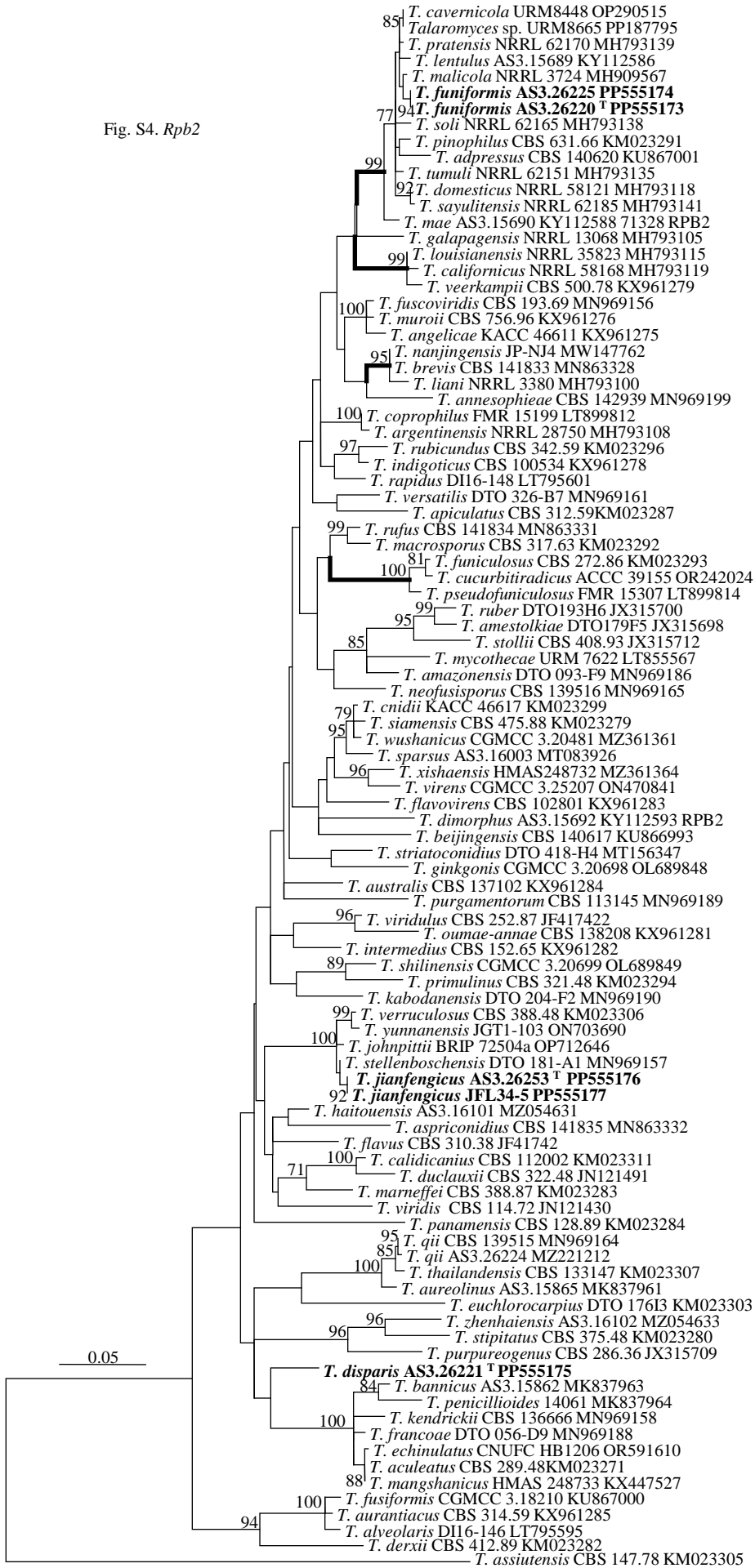

Supplement: Supplemental Information 5 — Bootstrap percentages over 70% derived from 1000 replicates are indicated at the nodes. New species are indicated in boldface. Bar = 0.05 substitutions per nucleotide position. [file peerj-12-18253-s005.pdf]

Fig. S5. ITS1-5.8S-ITS2

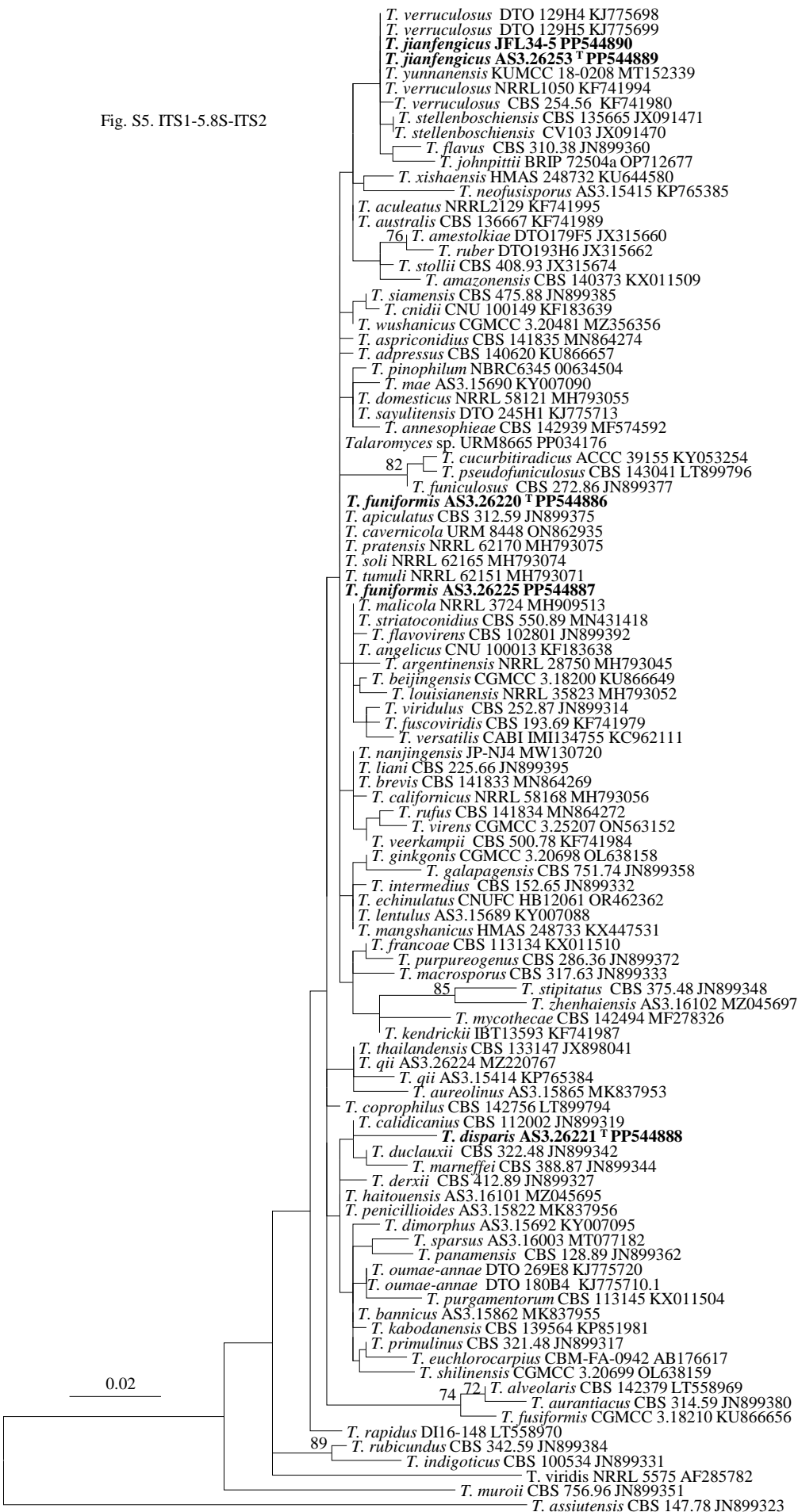

Supplement: Supplemental Information 6 — Bootstrap percentages over 70% derived from 1000 replicates are indicated at the nodes. New species are indicated in boldface. Bar = 0.02 substitutions per nucleotide position. [file peerj-12-18253-s006.pdf]
